# Lead‐Related and Procedural Outcomes of His Bundle Pacing Versus Left Bundle Branch Area Pacing in Patients Undergoing Atrioventricular Node Ablation: A Systematic Review and Meta‐Analysis

**DOI:** 10.1002/joa3.70419

**Published:** 2026-07-14

**Authors:** Norma N. Gamarra‐Valverde, Bezalel Hakkeem, Joseph E. Marine, Jhordan Chilon‐Cerdan, Adriana C. Mares, Juliana Giorgi

**Affiliations:** ^1^ Universidad Peruana Cayetano Heredia Facultad de Medicina Alberto Hurtado Lima Peru; ^2^ Government Medical College Kozhikode Kozhikode Kerala India; ^3^ Division of Cardiology, Department of Medicine Johns Hopkins University School of Medicine Baltimore Maryland USA; ^4^ Universidad Peruana de Ciencias Aplicadas Lima Peru; ^5^ Georgetown University School of Medicine Washington DC USA; ^6^ Hospital Sírio Libanês São Paulo São Paulo Brazil

## Abstract

**Background:**

In patients undergoing atrioventricular node ablation (AVNA), permanent pacing is required, making lead performance and procedural durability important. His bundle pacing (HBP) and left bundle branch area pacing (LBBAP) are physiologic pacing strategies used in this setting, but comparative evidence remains limited.

**Methods:**

We searched PubMed/MEDLINE and Embase through January 31, 2026, for comparative studies of HBP versus LBBAP in adults undergoing AVNA. The primary outcome was chronic lead‐related dysfunction, defined as clinically significant chronic capture‐threshold elevation. Secondary outcomes were AVNA procedural duration and atrioventricular nodal reconduction requiring repeat ablation. Random‐effects models generated pooled risk ratios (RRs) or mean differences (MDs). Certainty was assessed using GRADE.

**Results:**

Three observational studies comprising 434 patients were included. Two studies (*n* = 270) contributed to chronic lead‐related dysfunction, which occurred more often with HBP than LBBAP (RR 15.43, 95% CI 2.80–85.04); the estimate was imprecise because of sparse events. Two studies (*n* = 262) contributed to procedural duration, which was longer with HBP (MD 11.33 min, 95% CI 6.64–16.02; *I* ^2^ = 0%). Two studies (*n* = 262) contributed to atrioventricular nodal reconduction, which was more frequent with HBP (RR 19.34, 95% CI 2.60–143.61; *I* ^2^ = 0%). Certainty was low for procedural duration and very low for the other outcomes.

**Conclusions:**

Among patients undergoing AVNA, LBBAP was associated with more favorable lead‐related and procedural outcomes than HBP across available observational studies. Because evidence is limited by small cohorts, sparse events, and nonrandomized designs, these findings should be considered hypothesis‐generating.

**Trial Registration:**

This systematic review was registered in the PROSPERO database (registration number: CRD420261424072)

## Graphical abstract

In patients undergoing atrioventricular node ablation, left bundle branch area pacing was associated with fewer chronic high‐threshold events, shorter procedural duration, and less atrioventricular nodal reconduction than His bundle pacing. Given the observational nature of available studies, these findings should be considered hypothesis‐generating.

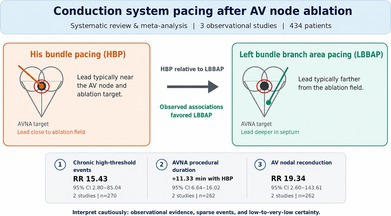

## 1 Introduction

Atrioventricular node ablation (AVNA) is an established treatment strategy for selected patients with symptomatic, drug‐refractory atrial fibrillation or other atrial tachyarrhythmias when rhythm‐control interventions are unsuccessful or not tolerated and pharmacological rate control remains inadequate [1, 2]. Because AVNA intentionally creates permanent atrioventricular block, patients become dependent on cardiac pacing, making the choice of pacing strategy an important determinant of procedural safety and long‐term device performance.

Conventional right ventricular pacing may result in ventricular desynchrony and pacing‐induced cardiomyopathy, particularly in patients with a high ventricular pacing burden or pre‐existing left ventricular dysfunction [1, 3]. Conduction system pacing (CSP), including His bundle pacing (HBP) and left bundle branch area pacing (LBBAP), has therefore emerged as a physiologic alternative intended to preserve ventricular activation through the native His–Purkinje system [1, 2, 3, 4].

Although HBP and LBBAP share the goal of physiologic ventricular activation, they differ substantially in target location and procedural anatomy. HBP typically captures the proximal His bundle near the atrioventricular node, whereas LBBAP targets the distal conduction system within the interventricular septum [5, 6, 7]. In patients undergoing AVNA, this anatomical distinction may be clinically relevant because the HBP lead and the ablation target may be located in close proximity. Ablation energy delivered near the His region could plausibly affect capture thresholds or lead stability, whereas LBBAP is generally positioned deeper within the septum and farther from the ablation field [8, 9, 10, 11].

The clinical landscape of conduction system pacing has also evolved rapidly. HBP represented the first widely adopted physiologic pacing strategy, but contemporary practice has increasingly shifted toward LBBAP because of its procedural reproducibility and stable electrical performance. In parallel, newer approaches to HBP, including distal His bundle pacing and deep septal distal His bundle pacing, have been described and may reduce some of the limitations associated with conventional proximal HBP [12, 13, 14]. However, these more recent HBP techniques were not consistently represented in the comparative studies currently available.

Several observational studies have compared HBP and LBBAP in patients undergoing AVNA and have reported differences in lead‐related and procedural outcomes [8, 9, 10]. Nevertheless, the evidence remains limited to nonrandomized studies with relatively small cohorts, heterogeneous outcome definitions, and variable follow‐up durations. A formal synthesis may therefore help clarify the consistency of available findings, quantify the magnitude and precision of reported associations, and define the certainty of evidence supporting these observations.

Therefore, we performed a systematic review and meta‐analysis of comparative studies evaluating HBP versus LBBAP in adults undergoing AVNA. Our objective was to synthesize the available evidence regarding lead‐related durability and procedural outcomes, while explicitly considering the limitations of the current evidence base.

## 2 Methods

This systematic review and meta‐analysis was conducted and reported in accordance with the Preferred Reporting Items for Systematic Reviews and Meta‐Analyses (PRISMA) statement [15] and guidance from the Cochrane Handbook for Systematic Reviews of Interventions [16]. The review was registered in the PROSPERO database on February 5, 2026 (registration number: CRD420261424072).

Given the absence of randomized trials in this specific procedural setting, we synthesized evidence from comparative observational studies evaluating His bundle pacing (HBP) versus left bundle branch area pacing (LBBAP) in adults undergoing atrioventricular node ablation (AVNA). A qualitative synthesis was planned for outcomes that could not be pooled because of heterogeneous definitions, inconsistent reporting, or insufficient extractable data.

### 2.1 Search Strategy and Data Extraction

A comprehensive systematic search was conducted in PubMed/MEDLINE and Embase from database inception through January 31, 2026. The search strategy combined controlled vocabulary terms and free‐text keywords related to atrioventricular node or junction ablation and conduction system pacing. Search terms included combinations of: “atrioventricular node ablation,” “AV node ablation,” “AV junction ablation,” “atrioventricular junction ablation,” “His bundle pacing,” “HBP,” “left bundle branch pacing,” “left bundle branch area pacing,” “LBBP,” and “LBBAP.”

No restrictions were applied by language, publication date, or study location. Reference lists of included studies and relevant review articles were manually screened to identify additional eligible reports.

### 2.2 Study Selection and Eligibility Criteria

All retrieved records were exported to reference management software, and duplicate records were removed before screening. Two investigators independently screened titles and abstracts, followed by full‐text review of potentially eligible articles. Disagreements were resolved by discussion and consensus. Studies were eligible if they met all of the following criteria: (1) included adult patients undergoing AVNA; (2) directly compared HBP with LBBAP; and (3) reported extractable quantitative data for at least one prespecified lead‐related or procedural outcome separately by pacing strategy.

Prospective and retrospective comparative observational studies were eligible. Studies were excluded if they did not involve AVNA, did not report outcomes separately for HBP and LBBAP, evaluated only a single pacing strategy without a comparator group, or were case reports, reviews, editorials, letters without original data, or conference abstracts without sufficient extractable information. In cases of potential cohort overlap from the same institution or study period, the most complete and clinically relevant dataset was selected.

### 2.3 Data Extraction

Two investigators (N.N.G.V and B.H) independently extracted data using a standardized extraction form. Extracted variables included study design, study period, sample size, patient characteristics, pacing strategy, follow‐up duration, procedural characteristics, lead‐related outcomes, AVNA procedural outcomes, and echocardiographic outcomes when available.

For each outcome, the number of contributing studies and patients was recorded separately because not all included studies reported all prespecified outcomes. When outcome definitions differed across studies, definitions were extracted exactly as reported and assessed for clinical comparability before quantitative pooling. Discrepancies in extracted data were resolved by discussion and consensus.

### 2.4 Endpoints

The primary outcome was chronic lead‐related dysfunction, operationalized for quantitative synthesis as clinically significant chronic pacing capture‐threshold elevation. Because included studies used different definitions of threshold elevation, pooling was restricted to studies with clinically comparable definitions of chronic high‐threshold events. Specifically, studies defining chronic high‐threshold events as capture threshold ≥ 2.0 V or ≥ 2.5 V at 0.5 ms were considered sufficiently comparable for quantitative synthesis. One study used a lower threshold definition of ≥ 1.0 V, which was considered clinically distinct from the higher‐threshold definitions used in the other studies. Therefore, this study was not included in the pooled analysis of chronic high‐threshold events and was summarized descriptively.

When reported, lead revision, lead deactivation, loss of effective conduction system capture, or clinically relevant threshold instability were extracted as additional lead‐related outcomes and summarized descriptively if definitions or reporting were not sufficiently comparable for pooling.

Secondary outcomes included AVNA procedural duration and atrioventricular nodal reconduction requiring repeat ablation. Acute lead‐related outcomes, including acute threshold rise during or immediately after ablation and exit block, were extracted when available but summarized descriptively because of heterogeneous reporting. Echocardiographic outcomes, including changes in left ventricular ejection fraction, were also extracted when available and summarized descriptively when quantitative pooling was not appropriate.

### 2.5 Quality Assessment

Two investigators (N.N.G.V and A.M) independently assessed the methodological quality of included studies using the Risk Of Bias In Non‐randomized Studies of Interventions (ROBINS‐I) tool [17]. The ROBINS‐I framework evaluates bias across seven domains: confounding, selection of participants, classification of interventions, deviations from intended interventions, missing data, measurement of outcomes, and selection of reported results.

Each study was categorized as having low, moderate, serious, or critical overall risk of bias according to ROBINS‐I guidance. Disagreements were resolved through discussion and consensus. Because all included studies were nonrandomized, particular attention was given to confounding, participant selection, and differences in follow‐up duration between pacing groups.

Given the limited number of included studies, formal assessment of publication bias using funnel plots or regression‐based methods was not performed.

### 2.6 Statistical Analysis

Dichotomous outcomes were pooled as risk ratios (RRs) with 95% confidence intervals (CIs), and continuous outcomes were pooled as mean differences (MDs) with 95% CIs. For all analyses, effect estimates were calculated as HBP relative to LBBAP; therefore, RR values greater than 1.0 indicate a higher risk of adverse events with HBP.

Random‐effects models were used for all pooled analyses because clinical and methodological heterogeneity was anticipated across observational studies. Dichotomous outcomes were pooled using the Mantel–Haenszel method. Continuous outcomes were pooled using inverse‐variance methods. Statistical heterogeneity was assessed using the *I* ^2^ statistic and interpreted in the context of clinical heterogeneity and the small number of included studies.

For dichotomous outcomes with zero events in one treatment arm, a continuity correction was applied as implemented in the meta‐analysis software. Because event counts were sparse and some comparisons included zero‐event cells, relative effect estimates were interpreted cautiously and considered exploratory. Outcomes reported as medians with interquartile ranges were summarized descriptively when reliable conversion to mean and standard deviation was not feasible.

Meta‐regression, subgroup analyses, and formal small‐study‐effect analyses were not performed because of the limited number of included studies. Sensitivity analyses using fixed‐effect models were performed to assess the directional robustness of pooled estimates.

Dichotomous outcome analyses and their corresponding forest plots were generated using Review Manager (RevMan) version 5.4 (Cochrane Collaboration). The forest plot for the continuous outcome of AVNA procedural duration was generated using R statistical software (R Foundation for Statistical Computing, Vienna, Austria) with the meta package, applying the same inverse‐variance random‐effects approach described above.

### 2.7 Certainty of Evidence Assessment

The certainty of evidence for each pooled outcome was evaluated using the Grading of Recommendations Assessment, Development and Evaluation (GRADE) framework [18]. Because all included studies were observational, the certainty of evidence was initially rated as low and was further downgraded when appropriate based on risk of bias, inconsistency, indirectness, imprecision, and publication bias. Imprecision was judged with particular attention to sparse event counts, wide confidence intervals, and zero‐event cells. Certainty of evidence was categorized as high, moderate, low, or very low and assessed separately for each pooled outcome.

## 3 Results

### 3.1 Study Selection

**FIGURE 1:**
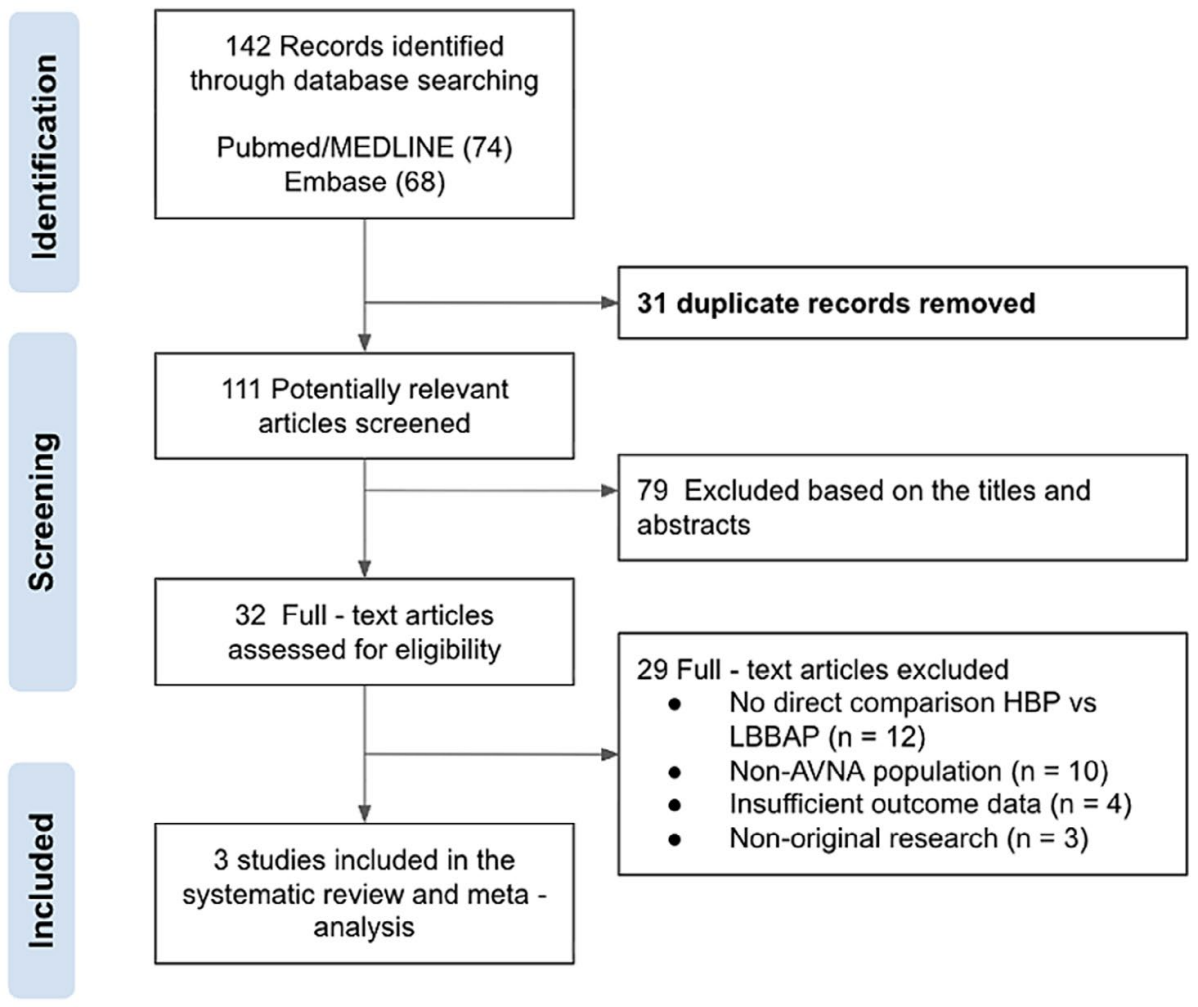
PRISMA flow diagram of study selection.

The systematic search identified 142 records across two electronic databases: PubMed/MEDLINE (*n* = 74) and Embase (*n* = 68). After removal of 31 duplicate records, 111 unique records underwent title and abstract screening. Of these, 32 articles were selected for full‐text review. Twenty‐nine full‐text articles were excluded because of absence of a direct comparison between HBP and LBBAP, non‐AVNA populations, insufficient outcome reporting, or non‐original research. Ultimately, three comparative observational studies comprising 434 patients were included in the systematic review and contributed data to at least one quantitative or descriptive analysis (Figure 1).

### 3.2 Study Characteristics

The three included studies enrolled a total of 434 patients, including 202 treated with HBP and 232 treated with LBBAP. Baseline clinical and procedural characteristics are summarized in Table 1. One study was prospective and comparative, one used propensity score matching, and one was a retrospective single‐center cohort. Sample sizes ranged from 98 to 172 patients.

**TABLE 1:** Baseline clinical and procedural characteristics of included studies comparing his bundle pacing and left bundle branch area pacing in patients undergoing atrioventricular node ablation.

Study	HBP (*n*)	LBBAP (*n*)	Mean age (years)		Female (%)		Baseline LVEF (%)		Baseline QRS (ms)		Backup RV lead used (%)		Acute capture threshold (V at pulse width reported)		Follow‐up (years)		High‐threshold definition
			HBP	LBBAP	HBP	LBBAP	HBP	LBBAP	HBP	LBBAP	HBP	LBBAP	HBP	LBBAP	HBP	LBBAP	
Chaumont et al. [8]	68	96	69.9 ± 12.3	75 ± 10.1	50	60	46 ± 15	49 ± 15	99 ± 17	107 ± 29	9	0	1.25 ± 0.6	0.75 ± 0.6	1.9 ± 1.5	1.2 ± 0.6	≥ 1 V
Cai et al. [10]	86	86	70.3 ± 10.4	70.5 ± 9.5	44.2	48.8	40.6 ± 14.8	41.2 ± 15	111.4 ± 32	111.7 ± 30.5	97.7	54.7	1.09 ± 0.7	0.46 ± 0.15	2.4 ± 1.0		≥ 2.0 V
Pillai et al. [9]	48	50	75.8 ± 7.9	77 ± 6.7	39.5	72	53.3 ± 9.4	53.1 ± 10.4	97.9 ± 24	102.7 ± 20.5	58	2	1.29 ± 1.03	0.68 ± 0.27	3 ± 1.4	1.0 ± 0.7	≥ 2.5 V

*Note:* Values are presented as mean ± standard deviation or percentage as reported in the original publications. Capture thresholds are reported at the pulse width specified in each individual study.

Abbreviations: AF, atrial fibrillation; AVNA, atrioventricular node ablation; HBP, His bundle pacing; LBBAP, left bundle branch area pacing; LVEF, left ventricular ejection fraction; QRS, QRS duration on baseline 12‐lead electrocardiogram; RV, right ventricular.

Follow‐up duration differed across studies and, in some cohorts, was longer in the HBP group than in the LBBAP group, reflecting earlier adoption of HBP before wider implementation of LBBAP. Baseline clinical characteristics were generally comparable between pacing strategies within individual studies, although sex distribution and use of backup right ventricular leads differed across some cohorts. Definitions of chronic high‐threshold events also varied between studies, ranging from ≥ 1.0 V to ≥ 2.5 V. Because not all included studies reported each prespecified outcome, the number of contributing studies and patients differed across pooled analyses.

### 3.3 Risk of Bias

Risk of bias assessment using ROBINS‐I is shown in Table 2. Overall risk of bias was rated as moderate in two studies and serious in one study. The main source of bias was confounding inherent to nonrandomized study designs. Additional concerns included participant selection and differential follow‐up duration between pacing groups. Outcome measurement was considered low risk across studies because the main outcomes were objective procedural or device‐related endpoints.

**TABLE 2:** Risk of bias assessment using ROBINS‐I.

Study	Confounding	Selection	Classification	Deviations	Missing data	Outcome measurement	Selective reporting	Overall risk
Chaumont [8]	Moderate	Moderate	Low	Low	Moderate	Low	Low	Moderate
Cai [10]	Moderate	Moderate	Low	Low	Moderate	Low	Low	Moderate
Pillai [9]	Serious	Moderate	Low	Low	Moderate	Low	Low	Serious

### 3.4 Primary Outcome: Chronic Lead‐Related Dysfunction (Chronic High‐Threshold Events)

Two studies comprising 270 patients contributed data to the pooled analysis of chronic lead‐related dysfunction, operationalized as chronic high‐threshold events. These studies were Cai et al. and Pillai et al., which used threshold definitions of ≥ 2.0 V and ≥ 2.5 V, respectively. The Chaumont et al. study used a lower threshold definition of ≥ 1.0 V and was therefore summarized descriptively rather than pooled for this outcome.

**FIGURE 2:**
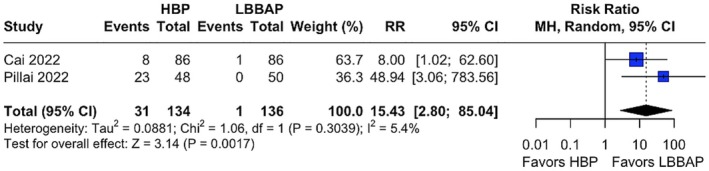
Forest plot of chronic high‐threshold events comparing His bundle pacing and left bundle branch area pacing. Two studies comprising 270 patients contributed to this analysis.

Among the two pooled studies, chronic high‐threshold events occurred in 31 of 134 patients in the HBP group and in 1 of 136 patients in the LBBAP group. HBP was associated with a higher relative risk of chronic lead‐related dysfunction compared with LBBAP (RR 15.43; 95% CI 2.80–85.04; *p* = 0.0017; *I* ^2^ = 5.4%) (Figure 2). However, this estimate should be interpreted cautiously because event counts were sparse, and one comparison included a zero‐event cell.

### 3.5 Secondary Outcomes

#### 3.5.1 AVNA Procedural Duration

**FIGURE 3:**
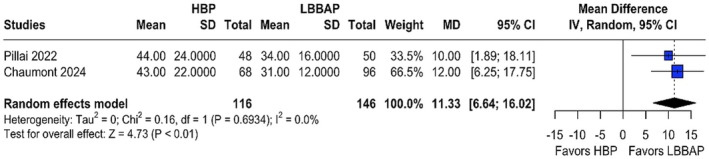
Forest plot of AVNA procedural duration comparing His bundle pacing and left bundle branch area pacing. Two studies comprising 262 patients contributed to this analysis.

Two studies comprising 262 patients reported AVNA procedural duration as mean ± standard deviation and were included in the pooled analysis. Procedural duration was longer in the HBP group than in the LBBAP group (MD 11.33 min; 95% CI 6.64–16.02; *p* < 0.01; *I* ^2^ = 0%) (Figure 3). Although statistical heterogeneity was not observed, interpretation of heterogeneity is limited by the small number of contributing studies.

#### 3.5.2 Atrioventricular Nodal Reconduction/Need for Repeat AVNA

**FIGURE 4:**
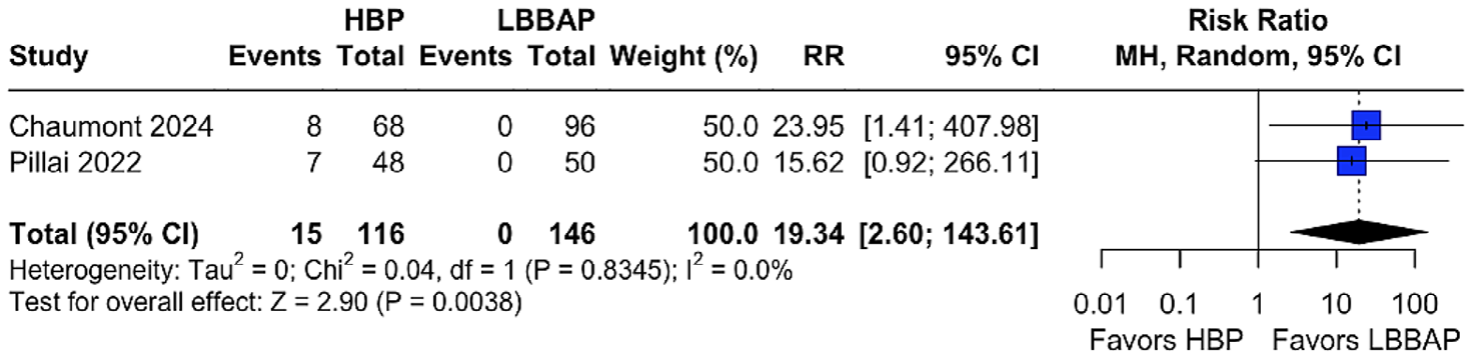
Forest plot of atrioventricular nodal reconduction requiring repeat ablation comparing His bundle pacing and left bundle branch area pacing. Two studies comprising 262 patients contributed to this analysis.

Two studies comprising 262 patients contributed data to the pooled analysis of atrioventricular nodal reconduction requiring repeat ablation. Events occurred in 15 of 116 patients in the HBP group and in 0 of 146 patients in the LBBAP group. HBP was associated with a higher relative risk of atrioventricular nodal reconduction compared with LBBAP (RR 19.34; 95% CI 2.60–143.61; *p* = 0.0038; *I* ^2^ = 0%) (Figure 4). Because all events occurred in the HBP group and the LBBAP arm had zero events, the magnitude of the pooled relative estimate is imprecise and should be considered exploratory.

#### 3.5.3 Acute Lead‐Related Outcomes and Echocardiographic Outcomes

Acute lead‐related outcomes, including acute threshold rise during or immediately after AVNA and exit block, were variably defined and inconsistently reported across studies, precluding quantitative pooling. Overall, acute lead‐related instability was reported more frequently with HBP than with LBBAP, but the available data were insufficient for a pooled estimate.

Echocardiographic outcomes, including changes in left ventricular ejection fraction, were also heterogeneously reported with variable follow‐up duration and incomplete modality‐specific data. Across the available studies, conduction system pacing was generally associated with preserved or improved left ventricular systolic function, but there was no consistent evidence allowing a reliable pooled comparison between HBP and LBBAP for echocardiographic outcomes.

### 3.6 Certainty of Evidence (GRADE)

The certainty of evidence for each pooled outcome is summarized in Table 3. Certainty was rated as very low for chronic high‐threshold events and atrioventricular nodal reconduction because of observational study designs, risk of bias, sparse event counts, zero‐event cells, and wide confidence intervals. Certainty was rated as low for AVNA procedural duration. Overall, the available evidence should be interpreted as hypothesis‐generating rather than definitive.

**TABLE 3:** Certainty of Evidence (GRADE) Assessment.

Outcome	Study design	Risk of bias	Inconsistency	Indirectness	Imprecision	Publication bias	Certainty of evidence
Chronic high‐threshold events	Observational	Serious	Not serious	Not serious	Serious (wide CI, sparse events)	Not assessable	Very Low
AVNA procedural duration	Observational	Serious	Not serious	Not serious	Not serious	Not assessable	Low
AV nodal reconduction	Observational	Serious	Not serious	Not serious	Serious (sparse events, wide CI)	Not assessable	Very Low

## 4 Discussion

In this systematic review and meta‐analysis of three comparative observational studies including 434 patients undergoing AVNA, we synthesized the available evidence comparing lead‐related and procedural outcomes between HBP and LBBAP. The main findings were that, across the limited available data, HBP was associated with more chronic high‐threshold events, longer AVNA procedural duration, and more frequent atrioventricular nodal reconduction requiring repeat ablation than LBBAP. However, these findings should be interpreted cautiously because the evidence was derived exclusively from nonrandomized studies, the number of contributing studies was small, event counts were sparse, and certainty of evidence was low or very low.

The purpose of this meta‐analysis was not to establish a practice‐changing superiority claim for LBBAP over HBP. Rather, it was to provide a focused quantitative synthesis of a small and fragmented comparative literature, estimate the precision of reported associations, and formally assess the certainty of available evidence. Although the individual studies included in this review had already suggested more favorable lead‐related and procedural outcomes with LBBAP, pooling the available data helps demonstrate that the direction of effect was broadly consistent across cohorts while also emphasizing the substantial uncertainty surrounding the magnitude of these estimates.

The observed association between HBP and chronic high‐threshold events is biologically plausible but should not be overinterpreted. In conventional HBP, the pacing lead is commonly positioned near the atrioventricular node and therefore close to the AVNA ablation target. This proximity may increase the risk of acute or delayed changes in capture threshold, either through direct thermal effects, local tissue injury, or subsequent fibrotic remodeling. In contrast, LBBAP is typically delivered deeper within the interventricular septum and farther from the ablation field, which may help preserve lead stability during and after AVNA. Nevertheless, because the pooled estimate was based on two studies and sparse events, the relative risk should be viewed as an exploratory measure rather than a definitive estimate of treatment effect.

Similarly, the association between HBP and longer procedural duration or atrioventricular nodal reconduction may reflect anatomic and procedural considerations. When a His bundle lead is located near the intended ablation site, operators may be more cautious with lesion delivery to avoid lead injury or threshold rise. This could plausibly prolong the procedure or reduce the durability of atrioventricular block. By contrast, the spatial separation between the LBBAP lead and the ablation target may allow more straightforward lesion delivery. However, this interpretation remains mechanistic and hypothesis‐generating because procedural technique, operator experience, ablation strategy, and follow‐up duration varied across studies.

An important consideration is that the HBP approach represented in the included comparative studies likely reflects earlier‐generation or conventional proximal HBP techniques. In particular, many HBP leads in the available cohorts were implanted before contemporary distal HBP strategies became more widely described. Newer techniques, including distal His bundle pacing from the right ventricle and deep septal distal His bundle pacing, may increase the distance between the pacing lead and the AVNA lesion set and could theoretically improve procedural safety, ablation durability, and threshold stability compared with conventional proximal HBP [12, 13, 14]. Therefore, the findings of this meta‐analysis should not be generalized uncritically to all contemporary HBP techniques.

This distinction is particularly relevant because conduction system pacing has evolved rapidly. LBBAP has been increasingly adopted in contemporary practice because of its procedural reproducibility and favorable electrical performance. As a result, the clinical relevance of directly comparing conventional HBP with LBBAP may be diminishing in some centers. In this context, the present study is best interpreted as a synthesis of available historical comparative evidence that helps explain the procedural and lead‐related factors contributing to the broader transition from HBP toward LBBAP, rather than as definitive evidence intended to mandate one strategy over another.

It is also important to recognize that HBP and LBBAP are not necessarily mutually exclusive strategies. In selected procedural scenarios, both leads may be implanted, with one lead serving as backup pacing to improve safety in patients expected to become pacing‐dependent after AVNA [19]. For example, an HBP lead and an LBBAP or ventricular backup lead may be connected to separate device ports to provide redundancy and protect against acute or delayed loss of capture. Such hybrid or backup strategies were not evaluated in the studies included in this meta‐analysis and therefore remain outside the scope of the present findings.

From a clinical standpoint, these results suggest that lead stability and the anatomic relationship between the pacing site and the ablation target should be considered during procedural planning for AVNA. The available evidence favors LBBAP for the specific lead‐related and procedural outcomes evaluated in this review, but the low to very low certainty of evidence prevents firm conclusions. Clinicians should therefore integrate these findings with patient characteristics, operator expertise, institutional experience, pacing indication, left ventricular function, and the feasibility of backup pacing strategies.

The findings also highlight important priorities for future research. Prospective multicenter studies using standardized definitions of chronic threshold elevation, acute threshold rise, lead revision, loss of conduction system capture, and atrioventricular nodal reconduction are needed. Future studies should also report outcomes separately for conventional proximal HBP, distal HBP, deep septal distal HBP, and LBBAP. Given the increasing adoption of LBBAP in contemporary practice, future comparative studies may be especially informative if they evaluate LBBAP against biventricular pacing in patients undergoing AVNA, particularly those with mildly or moderately reduced left ventricular ejection fraction.

Overall, the present meta‐analysis suggests that LBBAP is associated with more favorable lead‐related and procedural outcomes than HBP among patients undergoing AVNA in the currently available observational literature. However, because the evidence is limited by small study numbers, nonrandomized designs, sparse events, and evolving pacing techniques, these findings should be considered hypothesis‐generating and interpreted with caution.

## 5 Limitations

This meta‐analysis has several limitations. First, the evidence base was small, with only three comparative observational studies comprising 434 patients, and each pooled outcome was informed by only two studies. Therefore, the robustness and generalizability of the pooled estimates are limited.

Second, all included studies were nonrandomized, which restricts causal inference and introduces the possibility of selection bias, residual confounding, and center‐ or operator‐specific effects. Although baseline characteristics were generally comparable within individual studies, unmeasured differences in patient selection, implantation strategy, ablation technique, and operator experience may have influenced the observed outcomes.

Third, event counts were low for several outcomes, and some comparisons included zero events in the LBBAP arm. As a result, relative effect estimates were large and accompanied by wide confidence intervals. These estimates are therefore imprecise and should be interpreted as exploratory rather than definitive. In particular, the magnitude of the risk ratios may be unstable because of sparse events and the use of continuity correction in zero‐event comparisons.

Fourth, follow‐up duration differed between pacing strategies in some cohorts, with longer follow‐up in HBP groups in certain studies. This may have increased the opportunity to detect chronic lead‐related events in HBP patients and may have affected cumulative event rates.

Fifth, definitions of chronic threshold elevation varied across studies. The pooled analysis of chronic lead‐related dysfunction included studies using thresholds of ≥ 2.0 V and ≥ 2.5 V at 0.5 ms, whereas the study using a lower threshold definition of ≥ 1.0 V was not pooled for this outcome because it was considered clinically distinct. Although this approach improved clinical comparability within the pooled analysis, it reduced the number of studies contributing to the primary outcome.

Sixth, the HBP cohorts included in the available comparative studies may largely reflect conventional or earlier‐generation HBP approaches. Contemporary techniques, including distal His bundle pacing and deep septal distal His bundle pacing, were not consistently represented. Therefore, the present findings should not be generalized uncritically to all modern HBP techniques.

Finally, heterogeneous reporting of acute lead‐related outcomes, echocardiographic outcomes, backup pacing strategies, and hybrid approaches precluded quantitative synthesis of several clinically relevant endpoints. Formal assessment of publication bias or small‐study effects was not feasible because of the limited number of included studies. Accordingly, the findings of this meta‐analysis should be considered hypothesis‐generating.

## 6 Conclusions

Among patients undergoing AVNA, LBBAP was associated with more favorable lead‐related and procedural outcomes than HBP in the available observational studies, including fewer chronic high‐threshold events, shorter procedural duration, and less atrioventricular nodal reconduction requiring repeat ablation. However, given the small number of nonrandomized studies, sparse events, and low to very low certainty of evidence, these findings should be interpreted cautiously and considered hypothesis‐generating.

## Author Contributions

N.N.G.‐V. conceptualized and designed the study, performed literature screening and data extraction, and wrote the original draft of the manuscript. B.H. contributed to study methodology, data extraction, and performed the statistical analysis. J.C.‐C. contributed to data verification, reference verification, methodological revision, and critical revision of the manuscript for important intellectual content. J.E.M., A.C.M., and J.G. contributed to data interpretation and critically revised the manuscript for important intellectual content. All authors reviewed and approved the final version of the manuscript.

## Funding

The authors have nothing to report.

## Ethics Statement

This study is a systematic review and meta‐analysis of previously published data and did not involve human participants or animals directly. Therefore, ethical approval was not required.

## Consent

Patient consent was not required as this study used aggregated data from previously published studies and did not involve individual patient data.

## Conflicts of Interest

The authors declare no conflicts of interest.

## Data Availability

The data that support the findings of this study are available from the corresponding author upon reasonable request.
